# Supplementary material for: Piriformospora indica Increases Resistance to Fusarium pseudograminearum in Wheat by Inducing Phenylpropanoid Pathway
Source: Int J Mol Sci. 2023 May 15;24(10):8797. doi: 10.3390/ijms24108797 (PMC10218886; doi:10.3390/ijms24108797)
Supplement: Supplementary file 1 [file ijms-24-08797-s001.zip › ijms-2381106-supplementary.pdf]

Table S1. Primers used for identification the key DEGs involved in lignin synthesis pathway

Gene ID		PRIMER NAME	PRIMER	PRODUCT SIZE
TraesCS2D02G377200	PAL	TraesCS2D02G377200_For	CTGTGACCAACCATGTCCAG	241
		TraesCS2D02G377200_Rev	TGACCATTGTTGTTGGTGCT	
TraesCS5A02G213900	CAD	TraesCS5A02G213900_For	TCTCCCCCTACAGCTTCTCA	235
		TraesCS5A02G213900_Rev	CAGGAGTCGACGAAGTAGCC	
TraesCS7A02G084600	COMT	TraesCS7A02G084600_For	TCCTGGTGCACGGTGTAGTA	214
		TraesCS7A02G084600_Rev	ACATCGTCCAGCTCCTTGTC	
TraesCS2B02G395400	CSE	TraesCS2B02G395400_For	GGACAGCCTATCCAAGGTGA	204
		TraesCS2B02G395400_Rev	GCGATGATGTCGGAGAAGAT	
TraesCS6B02G294100	4CL	TraesCS6B02G294100_For	AAGTTCAAGGGCTTCCAGGT	237
		TraesCS6B02G294100_Rev	GATCGCATGGGTGAAGTACA	
TraesCS7D02G239400	CCOACMT	TraesCS7D02G239400_For	AGCTCCGACAGATCACCAAC	213
		TraesCS7D02G239400_Rev	GGTCGAAGTACTCCCTGCTG	
TraesCS5B02G268300	CCR	TraesCS5B02G268300_For	GTTCCTTCGGGAATCCTAGC	229
		TraesCS5B02G268300_Rev	GGTACCAGCTCTCGTTGCTC	
AY635186	FpTub	FpTub-For	GGTCTCGACAGCAATGGTGTT	158
		FpTub-Rev	GCTTGTGTTTTTCGTGGCAGT	

Table S2. Primers used for identification the key DEGs involved in flavonoid synthesis pathway

Gene ID		PRIMER NAME	PRIMER	PRODUCT SIZE
TraesCS5D02G488700	CHS	TraesCS5D02G488700_For	GGATGTGCCTGTAGCCATCT	233
		TraesCS5D02G488700_Rev	CGAGGATGAAGAGCACACAA	
TraesCS5D02G489000	CHI	TraesCS5D02G489000_For	GGCGAGTTCGAGAAGTTCA	206
		TraesCS5D02G489000_Rev	GAGTGGGTGAAGAGGATGGA	
TraesCS7A02G333900	FOMT	TraesCS7A02G333900_For	GCTGCTCGAGGTCTACAAGG	243
		TraesCS7A02G333900_Rev	CAGTCGTGGAGGATCCACTT	
TraesCS2B02G103600	IFR	TraesCS2B02G103600_For	GCAGGGTTCTTGTTGTTGGT	227
		TraesCS2B02G103600_Rev	CACCACGTCTACCTGCTTCA	
TraesCS7D02G152300	ANR	TraesCS7D02G152300_For	GCCAGTCCGTCTTCCTCAT	230
		TraesCS7D02G152300_Rev	AGCACTCCGGAGAGGGTAGT	
TraesCS4D02G227300	UGT	TraesCS4D02G227300_For	GTGGAACTCCACGCTAGAGG	212
		TraesCS4D02G227300_Rev	CTCTTTCCTTGGCACTTTCG	

Table S3. Differentially expressed genes (DEGs) in different comparison group

number	comparison group	DEGs number	Up expression	Down expression
1	Fp vs. mock	31355	17859	13496
2	Piri vs. mock	641	147	494
3	Piri+Fp vs. mock	27516	17142	10374
4	Piri+Fp vs. Piri	20757	11467	9290
5	Piri+Fp vs. Fp	3941	2559	1382
